# 

**Published:** 1889-04

**Authors:** 


### CONTENTS:

Article I.—Treatment of Proximate Surfaces. By Safford G. Perry, D. D. S. . . . 529

" II.—Dental Treatment of Children By Dr. Garrett Newkirk. . . . . 542

" III.—Treating Roots By Dr. Louis Ottofy. . . 549

" IV.—Septic Root Canals. By Dr. W. E. Tucker. . 553

" V.—The Careful Finishing of Amalgam Fillings. By Geo. F. Cheney, D. D. S. . . . 556

" VI.—Immediate Insertion of an Artificial Lower Jaw After Removal of the Natural One. . . 558

" VII.—Chloroform Administration . . . . . 561

" VIII.—Dental Inlaying With Porcelain. . . . 562

### EDITORIAL.

The Dental Department of Iowa University. . . . . 568

The International Tooth Crown Company *versus* The Dentists of the United States. . . . . 571

### BIBLIOGRAPHICAL.

Harris' Principles and Practice of Dentistry. . . . . 574

### MONTHLY SUMMARY.

What Shall We Eat? . . . . . . . . . . 576

